# G-Quadruplexes in c-MYC Promoter as Targets for Cancer Therapy

**DOI:** 10.3390/biomedicines11030969

**Published:** 2023-03-21

**Authors:** Bárbara Bahls, Israa M. Aljnadi, Rita Emídio, Eduarda Mendes, Alexandra Paulo

**Affiliations:** Faculty of Pharmacy, Research Institute for Medicines (iMed.Ulisboa), Universidade de Lisboa, 1649-003 Lisbon, Portugal

**Keywords:** G-quadruplexes, *c-MYC*, cancer, small molecules, G4 stabilizers

## Abstract

Cancer is a societal burden demanding innovative approaches. A major problem with the conventional chemotherapeutic agents is their strong toxicity and other side effects due to their poor selectivity. Uncontrolled proliferation of cancer cells is due to mutations, deletions, or amplifications in genes (oncogenes) encoding for proteins that regulate cell growth and division, such as transcription factors, for example, *c-MYC*. The direct targeting of the c-MYC protein has been attempted but so far unsuccessfully, as it lacks a definite binding site for the modulators. Meanwhile, another approach has been explored since the discovery that G-quadruplex secondary DNA structures formed in the guanine-rich sequences of the *c-MYC* promoter region can downregulate the transcription of this oncogene. Here, we will overview the major achievements made in the last decades towards the discovery of a new class of anticancer drugs targeting G-quadruplexes in the *c-MYC* promoter of cancer cells.

## 1. Introduction

Cancer is one of the most prevalent diseases in the world, with nearly 18 million new cases and 10 million deaths in 2020 [1]. These numbers clearly indicate that, despite the existence of many different ways to treat cancer, such as surgery, radiation therapy, chemotherapy, immunotherapy, and targeted therapies, these treatments do not lead to a cure in most cases [2]. This can happen for different reasons, for example, due to the presence of metastasis, cancer heterogeneity, and resistance to traditional chemotherapy [3]. For these reasons, and also due to the side effects of radiation and chemotherapy, it is of the utmost importance to find more efficient therapies with fewer side effects.

Cancer encompasses a large group of disorders resulting from the alteration in the expression of several genes, which leads to abnormal cell growth [4]. One of such oncogenes is *c-MYC,* which is amplified in many solid tumors, including almost all serous ovarian, breast, and lung carcinomas [5]. In addition, the chromosomal translocation of *c-MYC* can occur in leukemias and lymphomas [6], and the activity of *c-MYC* can also be deregulated at the level of expression and stability of *c-MYC* mRNA and proteins [7]. Thus, for the above mentioned reasons, and also due to the fact that *c-MYC* encodes for a transcription factor that controls the transcription of many genes involved in cell cycle progression, cell growth, differentiation, apoptosis, metabolism, DNA replication, and mRNA maturation [7,8,9,10], c-MYC has for a long time been considered to be an attractive drug target [11]. The direct pharmacological inhibition of the c-MYC protein has not yet been achieved, probably because it lacks a definite binding site due to its flat structure. However, many other indirect strategies to target c-MYC have been attempted [12]. These strategies include blocking *c-MYC* transcription with BRD4, CDK7, and CDK9 inhibitors, blocking *MYC* mRNA translation by targeting mTOR and CPEB, and also targeting the regulators of c-MYC stability such as some kinases and/or deubiquitinases (USP28, USP36, USP7, PLK1, and AURKA). Moreover, targeting MYC-MAX complexes by using a decoy (OmoMYC), or targeting genes that exhibit synthetic lethality with the overexpression of MYC (for example, *CDK1* and *GLS*) have also been attempted [13,14].

Meanwhile, in the last two decades, another strategy to target c-MYC has been explored: to directly target the *c-MYC* gene at the transcriptional level. Here, we will summarize the achievements made so far to control the expression of this oncogene and inhibit cancer cell proliferation, with G-quadruplex-interactive small molecules.

## 2. G-Quadruplex Nucleic Acids as Drug Targets

**Figure 1:**
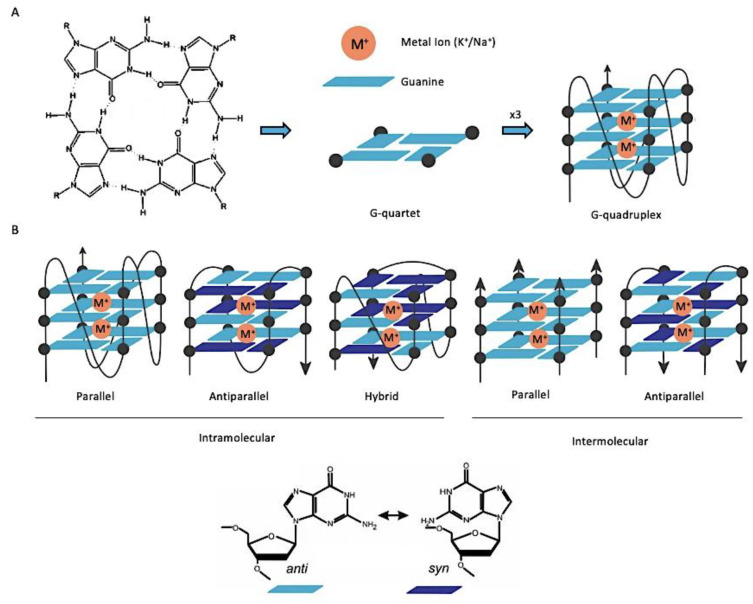
General structure and topologies of G-quadruplexes (G4). (**A**) Representation of a G-quartet stabilized by Hoogsteen hydrogen bonds (**left**). Schematic representation of a G-quadruplex additionally stabilized by *π*-*π* interactions and monovalent cations (**right**). (**B**) Schematic illustration of different intra- and intermolecular G4 topologies with different guanosine conformations (*syn* and *anti*).

G-quadruplexes (G4) are secondary structures formed from DNA or RNA sequences with at least four runs of consecutive guanines [15,16]. Hydrogen-bonding between four guanines forms planar square structures called G-tetrad or G-quartet, which in turn can stack on top of each other, in an arrangement stabilized by *π*-*π* interactions and monovalent cations, called G-quadruplexes (Figure 1A). G4s can be formed by single-stranded DNA (intramolecular) or by two or four different strands (intermolecular) [17]. The orientation of the glycosidic bonds (*syn* and *anti*), and the variations in the loop size and sequence, determine the topology (parallel, anti-parallel, or hybrid) and stability of the intramolecular quadruplex (Figure 1B) [17].

G4-forming sequences are not randomly distributed in the human genome. Computer analysis has revealed a prevalence of this type of sequence in the telomeres and promoter regions of proto-oncogenes and the involvement of G4s in the regulation of epigenetic, replication, transcription, and translation processes has been demonstrated by several studies [16,18,19,20]. These observations, combined with the fact that G4s can assume different topologies depending on the sequence and environmental factors inducing the folding [21], soon led researchers to hypothesize that G4 structures could be selectively targeted by small molecules. Over the years, many G4 ligands have been developed and studied in vitro and in vivo for their potential as drug leads to treat not only cancer but also other pathologies [22,23,24,25,26].

## 3. Regulation of *c-MYC* Transcription by G-Quadruplexes in Promoter Region

In normal circumstances, MYC expression is highly controlled, but in many cancer cells it is overexpressed, leading to tumor progression [13]. Consequently, changes in MYC’s expression are directly correlated to cancer development [9,27,28].

The *c-MYC* gene is part of a family composed of two other genes*, MYCN* and *MYCL*, that, like with *c-MYC*, encode for the proteins involved in cancer development [13,29]. Interestingly, this family includes two additional genes, *b-MYC* and *s-MYC*, that differ from the previous ones because they encode for proteins that suppress tumor growth [30,31]. 

**Figure 2:**
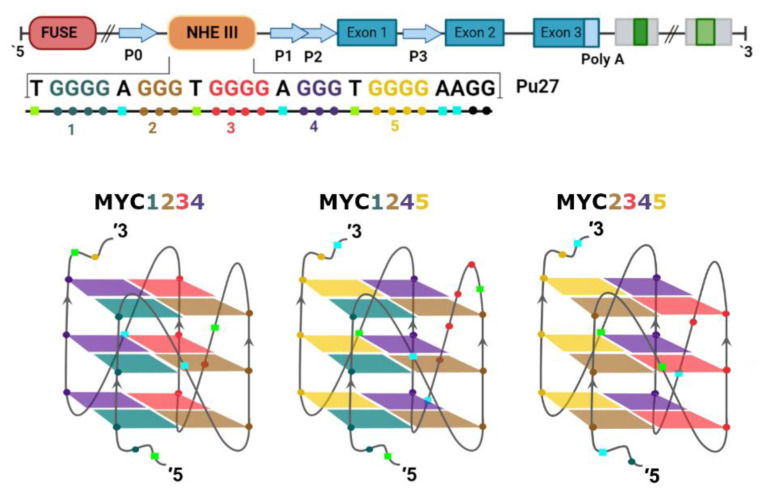
Pu27 *c-MYC* sequence (**top**) and representation of different guanine combinations to form different G-quadruplexes (**bottom**).

The *c-MYC* is regulated at transcriptional and post-transcriptional levels by several mechanisms [13]. This transcriptional regulation is complex and involves different elements, such as two transcription start locations, four promoters (P0, P1, P2, and P3), and the interaction with several DNA-binding proteins and regulatory elements. The most important promoters are P1 and P2 because they perform 75–90% of the transcriptions [32,33]. Upstream of the P1 promoter is a nuclease hypersensitivity element (NHE III1) that is the recognition region of transcription factor SP1, which activates *c-MYC* transcription. However, this is a GC-rich sequence called Pu-27, which can form intramolecular G4s that have been shown to silence *c-MYC* transcription [34,35,36,37]. This 27-nucleotide sequence has five runs of consecutive guanines, thus being able to fold into three different parallel G4s (Figure 2). However, the G4 formed by Runs 2, 3, 4, and 5 (MYC2345) was shown to be the more stable one [38], and for this reason it was considered to be the most relevant in the control of *c-MYC* transcription.

**Figure 3:**
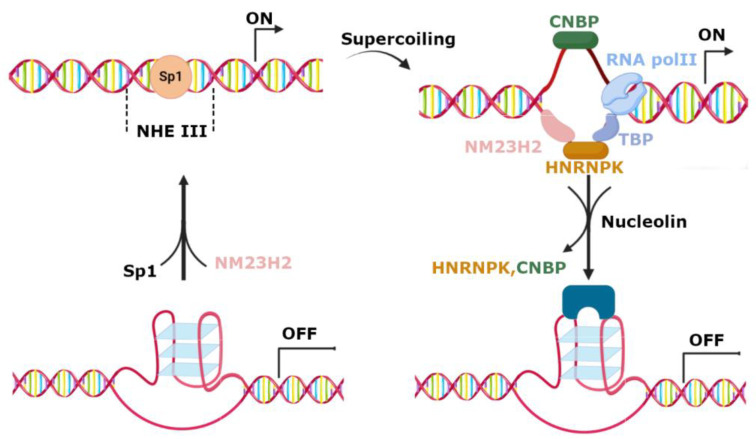
Schematic representation of *c-MYC* transcription regulation. G4 can downregulate the transcription by preventing the recognition of transcription factors. NM23H2 can unfold G4 leading to the transcriptional active DNA form. Single-strand DNA formation is induced by negative supercoiling, allowing for the binding of heterogeneous nuclear ribonucleoprotein K (HNRNPK) and the cellular nucleic acid binding protein (CNBP), and the posterior activation of *c-MYC* transcription. A negative regulation of this transcription occurs via the stabilization of G4 with nucleolin. Adapted from [24].

Active and silenced DNA secondary structures and consequent activation and deactivation of *c-MYC* transcription can be controlled by several proteins (Figure 3) [39]. Nucleolin regulates *c-MYC* transcription by promoting G4 formation and stabilization, leading to transcriptional arrest [40]. On the contrary, transcriptional activation is induced by the nucleoside diphosphate kinase NM23H2 protein [41] and poly ADP-ribose polymerase [42] that bind and unfold the c-MYC quadruplex. Single-strand DNA can be stabilized via the binding of heterogeneous nuclear ribonucleoprotein K (HNRNPK) and the cellular nucleic acid binding protein (CNBP), leading to the activation of *c-MYC* transcription [43]. 

## 4. *c-MYC* G4 Stabilizing Small Molecules with Anticancer Activity

Due to the interest in G4 structures as potential anticancer drug targets, a great number of small molecules has been studied to assess them as quadruplex stabilizing compounds [22]. These compounds typically consist of an aromatic core (usually polyaromatic or macrocyclic) with positively charged side chains, that can interact, respectively, with the G4 tetrads, their phosphate backbone, and/or with water molecules that are present in the grooves [22,44,45]. Many small molecules with antiproliferative activity in cancer cell lines also bind and strongly stabilize in in vitro DNA G4 structures of oncogene-promoter regions, suggesting a potential use of them as new anticancer drugs [46]. In this section, we will focus on the small molecules for which the anticancer mechanism of action has been suggested to be by targeting and stabilizing G4 in the promoter region of *c-MYC*.

### 4.1. Macrocycles 

Porphyrins are a family of N-heterocyclic molecules in which four pyrrole rings are connected, forming a macrocyclic compound. They provide a versatile base for several applications [47,48,49], such as chemosensors, photosensitizers [47], and antimicrobial agents [50], and as drugs in photodynamic therapy [51]. However, because of their photoactive properties, they can also induce harmful side effects.

**Figure 4:**
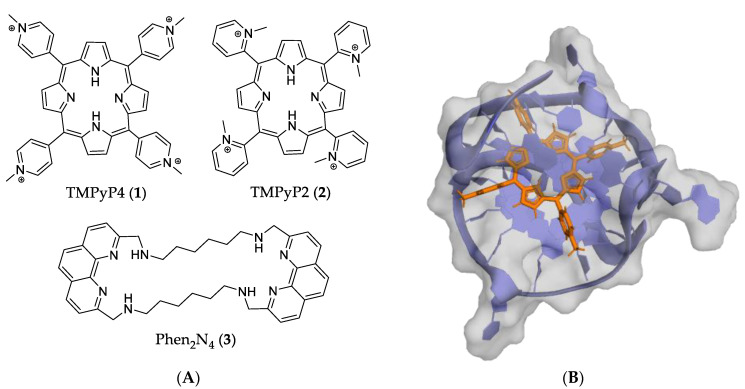
(**A**) Structures of porphyrin macrocycles **1** and **2** and macrocycle **3**. (**B**) PDB 2A5R representing porphyrin **1** in complex with a two-quartet c-MYC G4.

One of the most studied G4 ligands of this type is 5,10,15,20-tetrakis(N-methyl-4-pyridyl) porphyrin (TMPyP4) (**1**). TMPyP4 and its structural isomer TMPyP2 (**2**) (Figure 4A) can stabilize DNA G4 in different ways. The first is via external stacking with the outer layer of the guanine tetrads (Figure 4B) and the second is via binding to the diagonal and middle TTA loops [52,53,54]. Although both can stabilize the G4, Compound **2** has restricted rotation around the *meso* bond, which decreases its binding strength with the G-tetrads, justifying its low capacity to stabilize the G4 [37,54]. 

Furthermore, in vitro studies have shown that **1** can reduce telomerase activity, decrease *c-MYC* and *hTERT* expression, and inhibit cancer cell growth [55,56]. There are also a few in vivo studies that have focused on these compounds. These studies have shown that Compound **1** could decrease tumor growth and prolong animal survival compared with untreated controls and with Compound **2 [55].** However, **1** has shown poor selectivity for G4 structures compared to duplex DNA [57]. Moreover, additional studies have also shown that **1** is not able to discriminate between different G4 topologies [22]. 

Another class of macrocyclic compounds were studied by Carvalho et al. [58]. Different side chain lengths and NH content macrocyclic phenanthrolines were designed. The dimer phen_2_N_4_ (**3**) (Figure 4A) showed a good ability to stabilize both c-MYC G4 and telomeric G4 (21G), with a ΔTm of 17.2 and 20.3 °C, respectively, in a FRET melting assay. This experiment also showed that Compound **3** has selectivity towards G4 over duplex DNA. Moreover, the authors performed a fluorescent intercalator displacement (FID) assay, in which Compound **3** exhibited low values of EC_50_ (0.87 µM), which suggests a high binding affinity to c-MYC G4. Interestingly, an in vitro study demonstrated that Compound **3** can decrease the unwinding activity of Pif1 helicase. However, cytotoxicity assays resulted in no significant effect in the growth of HeLa cancer cells at low concentrations [58]. In another study, Compound **3** was shown to be highly cytotoxic (IC_50_ < 0.01 µM) for MCF-7 breast cancer cells [59]. 

### 4.2. Ligands with Four or More Fused Aromatic Rings

Indoloquinolines are tetracyclic aromatic compounds that have been extensively studied and have been shown to bind to several secondary structures of nucleic acids [60]. Moreover, several studies have shown that their selectivity and binding affinity toward G4s can change according to the side chains [23].

**Figure 5:**
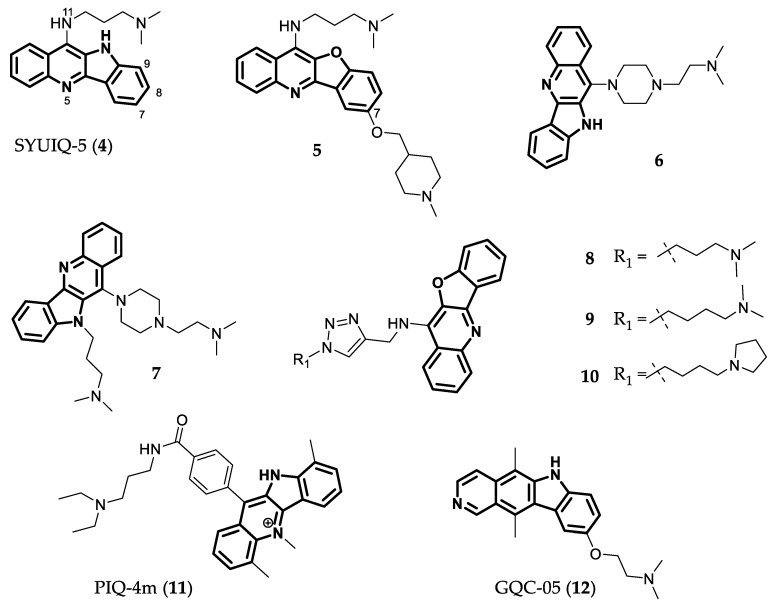
Structures of tetracyclic compounds: indoloquinolines **4**, **6**, **7,** and **11**, bioisosters **5** and **8–10,** and indoloisoquinoline **12**.

**Figure 6:**
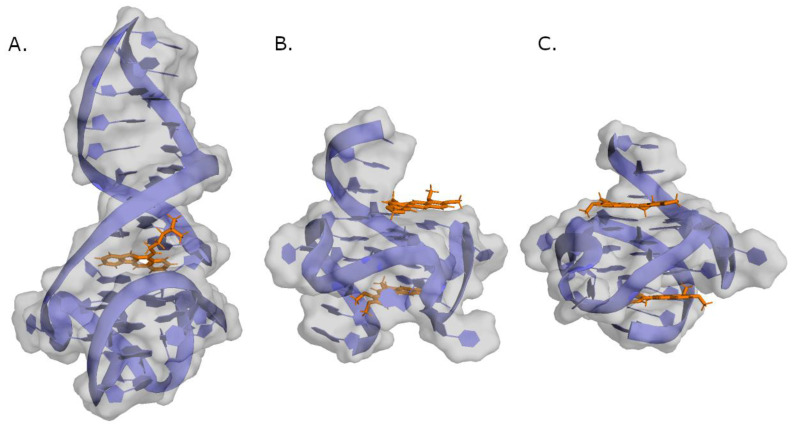
Representation of different compounds (colored sticks) in complex with a DNA structure (represented as cartoon and transparent surface). (**A**) Indoloquinoline **4** in complex with *c-*MYC G4 (PDB ID: 7PNG). (**B**) Conformer B of berberine in complex with c-MYC G4 (PDB ID: 7N7E). (**C**) Conformer A of berberine in complex with c-MYC G4 (PDB ID: 7N7D).

Taking this into consideration, Liu et al. [61] designed and analyzed four series of disubstituted indoloquinolines, with paired substitutions in Positions 7 and 11, 8 and 11, and 9 and 11, aiming to improve the anticancer activity of the lead SYUIQ-5 (**4**—Figure 5). Indoloquinoline **4** is a known binder of c-MYC G4, which intercalates between the 3′-outer tetrad of the G4 and a CG base pair (Figure 6A). Additionally, it establishes strong electrostatic interactions with guanine carbonyl groups, due to the positively charged protonated quinoline nitrogen of the tetracyclic indoloquinoline [62]. Moreover, this compound presented strong antiproliferative activity against several cancer cell lines, with IC_50_ values between 0.24 and 4.8 µM. It also reduced c-MYC transcription in cancer cells, which was suggested to occur by interfering with the interaction between NM23-H2 and c-MYC G4 [63].

After analyzing the di-substituted derivatives of **4**, Compound **5**, an indoloquinoline bioisoster with two tertiary amines in the side chains (Figure 5), was found to be the most promising one. In a FRET melting assay, the compound increased the melting temperature of c-MYC G4 in 26.6 °C and in a microscale thermophoresis (MST) assay showed a higher affinity to c-MYC G4 than to duplex DNA (K_D_^duplex^/K_D_^c-MYC^ = 8.0). Moreover, the compound can inhibit *c-MYC* transcription and cancer cell proliferation (IC_50_ of 4.7 µM) in Burkitt’s lymphoma (RAJI) cell line. Additionally, in vivo studies were performed in a RAJI xenograft model, and Compound **5** with a dosage of 30 mg/Kg for 2 weeks inhibited tumor growth, inducing a reduction in tumor volume of 27.4% [61]. Another study by Hurley et. al. analyzed indoloquinolines with a 11-piperazinyl substitution. The most promising compounds were **6** and **7** (Figure 5), which could stabilize the c-MYC G4, presenting ΔTm values of 7 and 17 °C in a CD melting assay. Additionally, the cytotoxicity in RAJI cell lines was tested, and both compounds also presented good IC_50_ values (2.3 and 3.1 µM, respectively) [64]. However, further experiments have suggested that the anticancer activity shown by these compounds cannot only be attributed to their capacity to stabilize c-MYC G4.

A different study demonstrated that three hybrids of triazole and indoloquinoline’s bioisoster (Compounds **8**, **9,** and **10**—Figure 5) that increased the melting temperature of c-MYC G4 between 13 and 22 °C could also downregulate *c-MYC* transcription and expression. They also showed high cytotoxicity for different cancer cell lines (IC_50_ = 0.02–5.53 µM) and lower cytotoxicity against non-malignant human cells. Additionally, Compound **10**, in an in vivo assay using a human lung cancer xenograft (A549 cell line), induced the significant inhibition of cancer cell proliferation (38%) and a decrease in tumor volume [65]. 

The N5-methylated indoloquinoline, PIQ-4m (**11**) (Figure 5), was shown to be a strong binder of c-MYC G4, with an EC_50_ of 1.7 µM in the thiazole orange (TO) displacement assay, and was also able to form a 1:2 ligand/G4 complex [66,67]. GQC-05 (**12**), an indoloisoquinoline derivative (Figure 5), is a well-known binder of c-MYC G4, inducing a ΔTm of 21 °C in a FRET melting assay. Moreover, it can downregulate the expression of this gene alone or in combination with other drugs, such as Navitoclax, a BCL-2 inhibitor [68,69,70,71]. 

**Figure 7:**
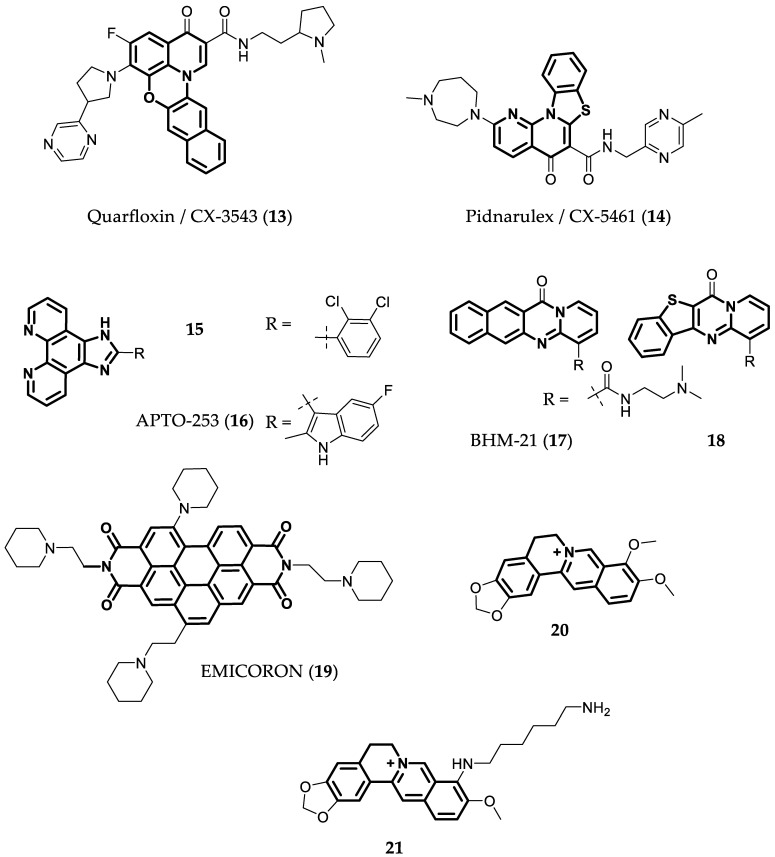
Structures of c-MYC G4 ligands with four or more fused aromatic rings.

Another class of compounds that can target DNA are fluoroquinolones [72]. Quarfloxin (CX-3543) (**13**) and Pidnarulex (CX-5461) (**14**) (Figure 7) are two fluoroquinolone derivatives that have been developed to preferentially target G4 nucleic acids, both inducing ΔTm > 15 °C in a FRET melting assay, for different G4s [73]. They entered into human clinical trials for the treatment of cancer [74,75,76]. Compound **13** reached a Phase II clinical study for the treatment of Neuroendocrine Carcinoma (NCT00780663), but it was abandoned due to poor clinical outcomes [76,77,78]. More recently, **14** entered in Phase I clinical trial for solid tumors withBRCA1/2, PALB2 or homologous recombination deficiency mutations (NCT04890613) [75]. The results of this Phase I study were recently released, demonstrating that the compound is well tolerated and that the observed reversion of PALB2 and BRCA2 mutations confirmed the proposed mechanism of action [79].

Compound **13** has higher selectivity to parallel G4 structures when compared to porphyrin **1** [24] and shows low IC_50_ values (around 2 µM) in cell viability assays with different cancer cell lines. Initially, the mechanism of action of 13 was thought to be through the binding to the G4 of the *c-MYC* promoter, but later it was discovered that it probably acts through binding to ribosomal DNA G4, inhibiting RNA-polymerase I activity and the formation of nucleolin/G4 complexes. This mechanism generates the redistribution of nucleolin into the nucleoplasm and, consequently, the inhibition of *c-MYC* transcription and cancer cell apoptosis [78]. There are several proposed mechanisms of action for Compound **14**, with the most acknowledged being the synthetic lethality due to the stabilization of G4 structures in DNA. This causes a loss of function of the replication fork and induces DNA breaks, leading to apoptosis, particularly in cells with deficiencies in the DNA damage repair mechanisms [79,80]. Moreover, another recent study has indicated that 14 also inhibits topoisomerase II activity [81].

It has been shown that phenanthroimidazole derivatives can act as anticancer agents through different mechanisms, including by binding to c-MYC G4. Wu et al. [82] analyzed six compounds with different substitutions in the benzene ring linked to the imidazole. The most promising derivatives were those with chlorine atoms. It was shown that Compound **15** (Figure 7) can downregulate the expression of *c-MYC* and stabilize the G4 in the promoter region of this gene (ΔTm = 4.4 °C in a FRET melting assay). This ligand also showed good antiproliferative activity against cancer cells (IC_50_ values around 1 µM) and moderate toxicity for non-cancer human keratinocyte HaCaT cells (IC_50_ = 16.8 µM). APTO-253 (**16**—Figure 7) is also a phenanthroimidazole derivative that entered Phase I clinical trials for patients with acute myelogenous leukemia (AML) or myelodysplastic syndrome (MDS) (NCT02267863) [83]. The first outcomes have been published, revealing that APTO-253 has been well tolerated at doses up to 150 mg/m^2^ [84]. Compound **16** is known to present antiproliferative activity against human colon leukemia, the non-small cell lung, and renal and prostate cancer cell lines by inducing Krüppel-like factor (KLF) tumor suppressors [85]. In a FRET melting assay, **16** showed time-dependent stabilization of G4s, with propensity to stabilize c-MYC G4, and it was more selective to G4 than Compounds **1** and **14** that were used as controls. Compound **16** is also able to induce cytotoxicity in AML and in different lymphoma cell lines, with IC_50_ values between 57 nM and 1.75 µM. It also decreases c-*MYC* expression at mRNA and protein levels [86].

Compound BMH-21 (**17**) and its congener **18** (Figure 7) are examples of other compounds with four fused aromatic rings that can efficiently bind to c-MYC G4, as was shown by NMR studies (ΔTm ~10 °C). They bind to the Pu22, a 22-mer sequence of Pu27, and inhibit lymphoma cell (SUDHL-4) growth with IC_50_ values below 1 µM. Compound **18** forms a 2:1 ligand/G4 complex, in which each molecule binds to a different part of G4 [87].

Another polyaromatic compound is EMICORON (**19**—Figure 7) , a perylene diimide derivative that causes telomere uncapping and binds to G4s through *π*-*π* interactions with the terminal G-tetrads. In addition, its side chains can bind to the grooves of G4 due to their positive charges. Studies have demonstrated that this compound can bind to the *c-MYC* and *BCL-2* oncogene promoter G4s (ΔTm = 16.4 and 15.4 °C, respectively, in a FRET melting assay) [88] and downregulate the expression of these oncogenes in cancer cells. Compound **19** presented good in vitro anticancer activity and in vivo studies with patient-derived xenografts of a metastatic lymph node of a colon carcinoma in a nude mouse showing a 64% inhibition of tumor growth when treated with **19** [89]. 

Berberine (**20**—Figure 7) is a natural product known to bind to G4 in a 2:1 stoichiometry as seen in the pdb structures of both conformers (7N7D and 7N7E)—Figure 6 [90]. This compound increased the melting temperature of c-MYC G4 by more than 6 °C in a CD study and it can induce cancer cell apoptosis in a dose-dependent manner. Additionally, in an in vivo assay with mice implanted with colon cancer cells (CT26), Compound **20** led to a significant decrease in tumor size after 14 days of treatment [91]. Berberine derivatives were also studied as c-MYC G4 ligands. One study showed that a 9-N-substituted derivative (**21**—Figure 7) could stabilize c-MYC G4 in a FRET melting assay, increasing the ΔTm of the complex to 29 °C. This compound could also inhibit the amplification of Pu27 in a PCR polymerase assay, with IC_50_ = 2 µM. Moreover, **21** promoted the total inhibition of HL60 cell proliferation at 5 and 10 µM and in an MTT assay presented IC_50_ = 4 µM in the NCI cell line [92].

### 4.3. Ligands with Three Fused Aromatic Rings

**Figure 8:**
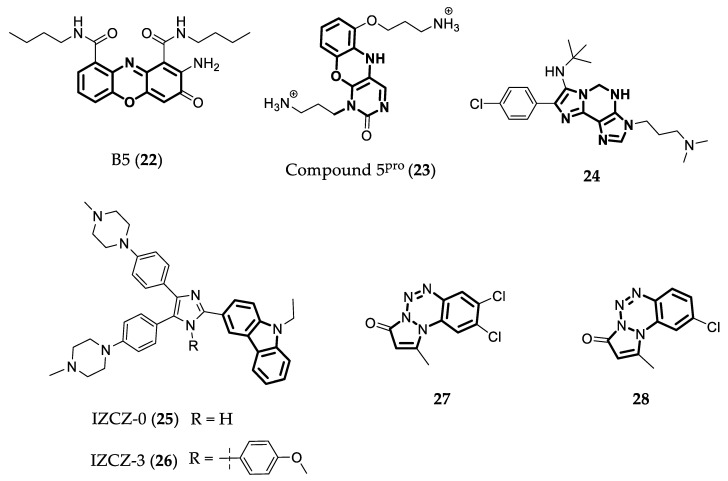
Structures of c-MYC G4 ligands with three fused aromatic rings.

Phenoxazine is a tricyclic molecule that is part of known antibiotic and antitumor agents [93,94]. Phenoxazone B5 (**22**—Figure 8) is a phenoxazine derivative discovered in an in silico study as being a promising G4 ligand. This compound showed antiproliferative activity and a larger inhibitory effect on *c-MYC* transcription (by 25–40%) in the Ramos cell line than in the CA46 cell line, in which the *c-MYC* transcription is no longer regulated by the NHEIII1 G4-forming sequence in the promoter. These observations strongly suggest that the compound targets the promoter c-MYC G4. Moreover, **22** also showed better inhibitory activity of c-MYC promoter amplification via Taq polymerase than of *BCl2*, *VEGF,* and *HIF-1α* promoters, which also have G4-forming sequences. However, by using CD and a molar equivalent of the ligand, the compound was unable to increase the melting temperature of c-MYC G4 [95]. In another study, focused on the lead 5^pro^ (Compound **23**—Figure 8), two other phenoxazines with different side chains were studied. However, these new compounds did not induce better variations in Tm of the G4 in a FRET melting assay (ΔTm= 0 and 9 °C, respectively), when compared to the lead compound (ΔTm = 18 °C) [93].

Another imidazole chemotype, imidazole-purines, that can bind to c-MYC G4 was studied by Pelliccia et al. [96]. They compared several compounds to determine which one had a better selectivity to G4s in *c-MYC* and *BCL2* gene promoters. Compound **24** (Figure 8) was the one with the ability to stabilize both G4s in a FRET melting assay (ΔTm = 12.8 °C for c-MYC G4 and 6.7 °C for BCL2 G4) and to downregulate *c-MYC* and *BCL2* expression by 66% and 67%, respectively, after the treatment of Jurkat cells (human leukemia cell line) with 25 µM of the compound for 24 h.

Based on the structure of IZCZ-0 (**25**—Figure 8), four compounds were synthesized and the most promising derivative was the tetra-aryl derivative IZCZ-3 (**26**—Figure 8). It exhibited stronger affinity to c-MYC G4 (ΔTm of 20 °C) in comparison to the other G4s. Additionally, it showed low IC_50_ values of cytotoxicity to SiHa, HeLa, Huh7, and A375 cancer cells (2.1–4.2 µM), and higher IC_50_ values to normal BJ fibroblasts and mesangial cell lines (IC_50_ = 15.6–15.9 µM). Furthermore, it showed anticancer activity in vivo against human cervical squamous cancer cells, inducing a reduction in tumor weight of 69, 64, and 57% after treatment with 20, 10, and 5 mg/kg of the compound, respectively [97].

Another class of compounds, the pyrazolo[1,2-a]benzo[1,2,3,4]tetrazin-3-one derivatives (PBTs), was studied to evaluate the interaction with c-MYC G4 and the regulation of this oncogene transcription. This study showed that the substitution in C8 and C9 (**27**) and only in C8 (**28**) with chlorine atoms (Figure 8) improves the G4 stabilizing effect of these derivatives (ΔTm = 4.0 and 1.9 °C, respectively). Moreover, molecular docking studies have predicted that these chlorine atoms interact with adenine 3 of the G4 [98]. These compounds also presented good antiproliferative activity against different cancer cell lines in an MTT assay, with IC_50_ values between 13.5 and 13.9 µM for Compound **26** and 17.7 and 20.5 µM for Compound **27** [99].

**Figure 9:**
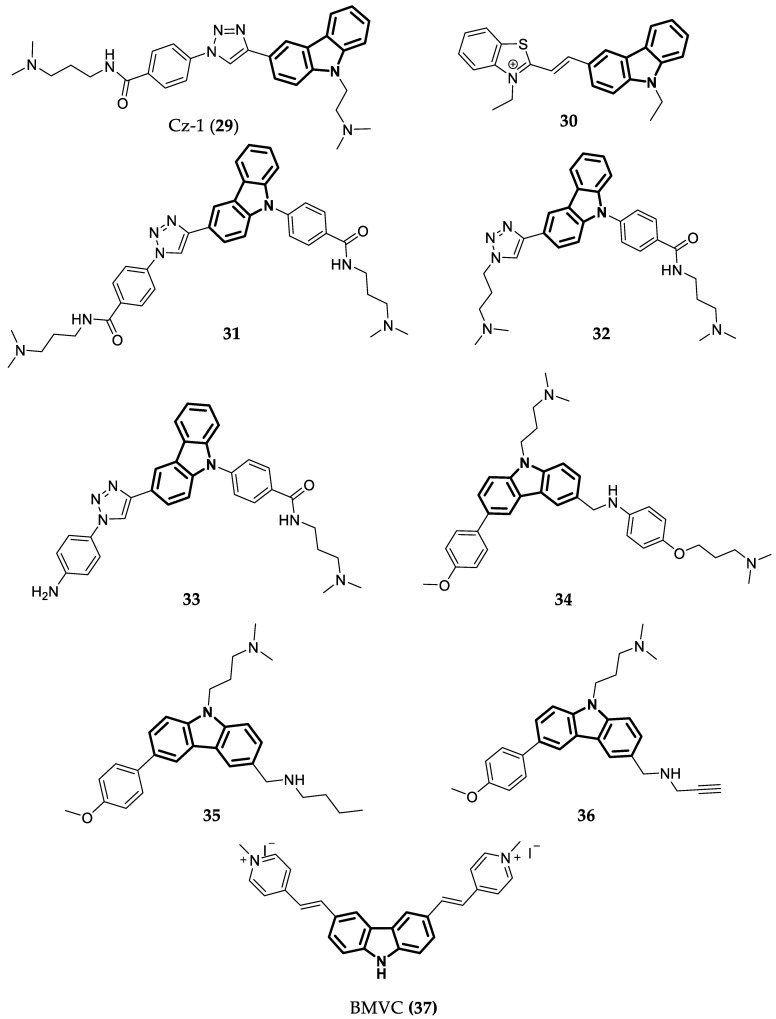
Structure of Carbazoles showing cancer cell antiproliferative activity and high capacity to stabilize *c-MYC* G4.

Due to their planar aromatic ring system, carbazoles are a good choice for quadruplex recognition. Several studies have analyzed the binding of carbazole derivatives to G4s. In one of these studies, a derivative named Cz-1 (**29**—Figure 9) induced the highest increase in melting temperature of c-MYC G4 permitted in the FRET melting assay (15.8 °C). In other words, **29** showed a good ability to stabilize c-MYC G4 [100]. Furthermore, this compound was shown to be selective to c-MYC G4 over duplex DNA, with K_D_ = 0.21 µM, and showed low values of IC_50_ in a cell proliferation assay with HeLa and HCT116 cells (3.4 µM and 3.2 µM, respectively) [100]. Another study conducted by Gluszynska et al. [101] also showed that carbazoles are potent G4 stabilizers. In this case, the carbazoles were also combined with triazoles and other pharmacophores. Interestingly, the most promising compound, **30** (Figure 9), was the only one with the carbazole and the benzothiazole moieties.

Several other hybrid molecules were developed, for example, carbazole-triazole **31**, **32,** and **33** (Figure 9). Compound **31** presented the best selectivity and a higher affinity to c-MYC G4 than the other two hybrids, with K_D_ = 0.17 µM. In a proliferation assay with HCT116 colon cancer cells, this compound showed an IC_50_ value of 2.1 µM and it was also proven that it was able to downregulate *c-MYC* transcription and to induce apoptosis in a dose-dependent manner [102]. Overall, Compound **31** was also better when compared to compound **29,** both in stabilizing the G4 and in the antiproliferative effect over HCT116 cancer cells.

In an additional study, a series of carbazole derivatives were designed and analyzed using a FRET melting assay to determine their selectivity for G4. The most promising was Compound **34** (Figure 9), showing a relatively high value of ∆Tm for G4-DNA (23.4 °C) and a lower value for double-stranded DNA. Compounds **35** and **36** (Figure 9) also presented good values of ∆Tm, but lower than those of **34.** Furthermore, **35** and **36** displayed significant cytotoxicity for cancer cell lines (IC_50_ = 2–6 µM) and insignificant values of cytotoxicity for human normal cells (IC_50_ *>* 50 µM) [103].

**Figure 10:**
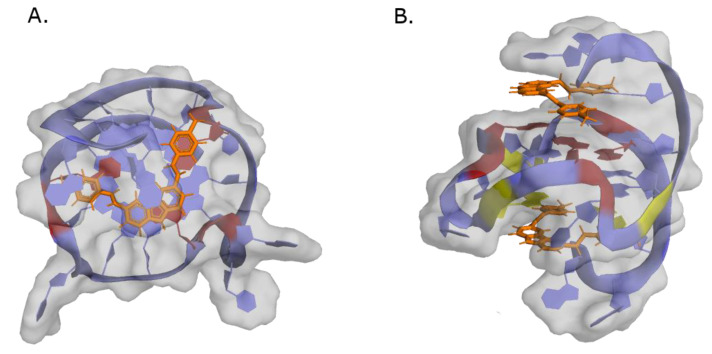
Representation of Compound **37** (colored sticks) in complex with a *c-MYC* G4 structure (cartoon). Guanines interacting with BMVC in top G-quartet at 5′ are colored red, and in yellow are the ones at 3′ G-tetrad. (**A**) represents the structure of the 1:1 complex (PDB ID: 6JJ0). (**B**) represents the structure of the 2:1 complex (PDB ID: 6O2L).

3,6-bis(1-methyl-4-vinylpyridinium carbazole diiodide (BMVC—**37**—Figure 9) is another carbazole that binds to c-MYC G4, forming 1:1 and 2:1 ligand/G4 complexes (Figure 10). BMVC changes its conformation to perform better binding to G4 and it perfectly matches three bases of the G-tetrads (Figure 10). Furthermore, Compound **36** showed K_D_ = 36 nM for its complex with c-MYC G4 and induced a significant increase in the thermal stability of this G4 in a CD assay. This compound was also shown to repress c-*MYC* expression in MCF-7 breast cancer cells at 4 and 10 µM concentrations [104].

### 4.4. Ligands with Two Fused Aromatic Rings

**Figure 11:**
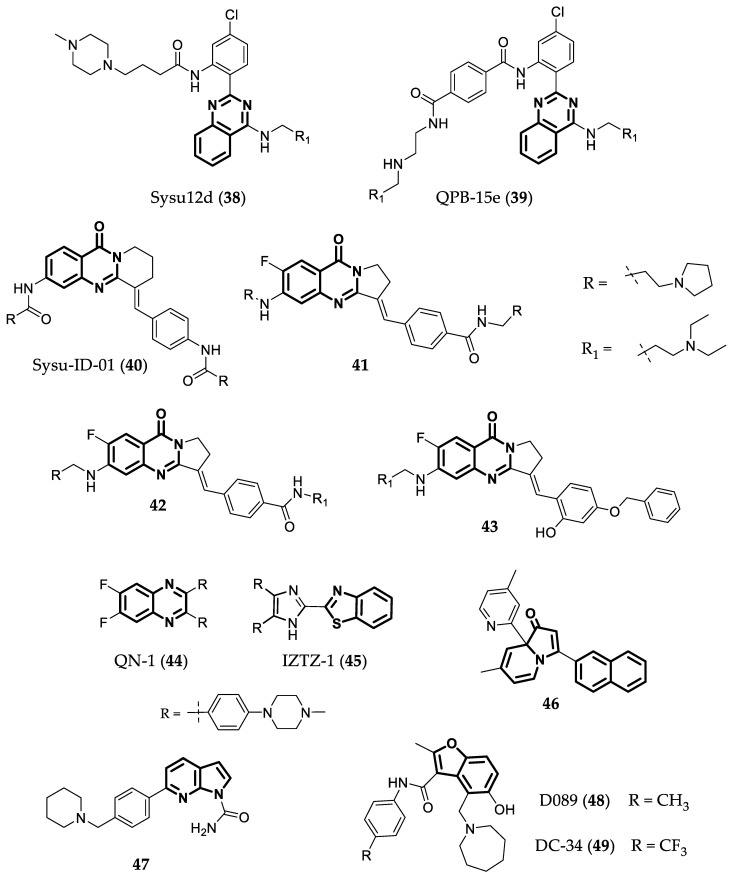
Structure of c-MYC G4 ligands with two fused aromatic rings.

Quinazolines are N-containing heterocyclic compounds that are widely used as therapeutic agents [105,106]. Sysu12d (**38**) (Figure 11) is a 2,4-disubstituted quinazoline derivative that can stabilize the c-MYC G4 in the Pu27 sequence [107], and downregulate RNA polymerase I through the disruption of the nucleolin–rDNA interaction. This compound also presented good antiproliferative activity against several cancer cell lines, with IC_50_ values between 3.1 and 6.3 µM [107]. Another study by Li et al. [108] showed that Compound **39** (Figure 11) could stabilize c-MYC G4 in a FRET melting assay (ΔTm = 23.7 °C) and reduce tumor growth by 49% (10 mg/kg) and 58% (20 mg/kg) in an in vivo assay using human liver cancer cell lines transplanted in a nude mouse. Sysu-ID-01 (**40**—Figure 11) is another quinazoline derivative that showed the ability to bind to NM23-H2, down- regulating the transcription and translation of *c-MYC*, but its binding to *c-MYC* G4 is not as potent (ΔTm = 9 °C) as to NM23-H2 [109]. Because of these results, more studies were conducted to improve compounds’ bioavailability and to study the effects of different substituents. The most promising compounds in this study, **41** and **42** (Figure 11), showed high and specific binding, as well as a stabilizing effect on c-MYC G4 (ΔTm = 12.1 and 12.9 °C, respectively), accompanied by the inhibition of this gene transcription. Additionally, in an in vivo anticancer assay, both compounds inhibited the proliferation of SiHa cells (squamus cell carcinoma) in a dose-dependent manner [110]. Another study by Wang et al. [111] found another isaindigotone derivative (Compound **43**—Figure 11) that could downregulate *c-MYC* transcription by disrupting the interaction between NM23-H2 and c-MYC G4. This compound presents weak binding to c-MYC G4 (ΔTm = 0.54 °C) but strong binding to the protein. Compound **43** can also induce cell cycle arrest, apoptosis, and SiHa cell proliferation arrest in a dose-dependent manner. In vivo studies in a SiHa xenograft mouse model were also performed. Compound **43** inhibited tumor growth by 48 and 65% at 2.5 and 5.0 mg/kg, respectively.

Quinoxaline or benzopyrazine compounds are naphthalene derivatives. Their main biological activities are antibacterial, antiviral, and antiparasitic, but they also present anticancer activity via different mechanisms [112,113]. Recently, Hu et al. [114] reported a new quinoxaline that can downregulate *c-MYC* transcription by targeting the G4s in the gene promoter. In this study, they synthesized 10 different quinoxalines with different side chains and several different electron-donating groups. Through structure–activity relationship studies, they concluded that the amino side chains were essential to the compounds binding to the G4 and extra positively charged amino substituents strengthened the interactions with c-MYC G4. The most promising compound was QN-1 (**44**) (Figure 11). It bound strongly to c-MYC G4 (K_D_ = 1.3 µM) and was selective to this G4 over other G4s with different topologies. Additionally, its structure is more “drug-like” than the other G4 ligands. It also presented low values of IC_50_ (0.7–0.9 µM) in a cell proliferation assay with different cancer cell lines and higher values compared to normal fibroblast cells (IC_50_ = 4.6 µM). Compound **44** also showed the ability to inhibit *c-MYC* transcription in a concentration-dependent manner and had weaker effects in other oncogenes’ transcriptions, which shows the preference to silence the *c-MYC* gene in the breast cancer 4T1 cell line. Moreover, they conducted an in vivo study to evaluate the ability of Compound **44** to inhibit tumor growth in a triple-negative breast cancer (TNBC) mouse model. At 2.5, 5, and 10 mg/kg, QN-1 significantly inhibited the cancer cell growth (TGI = 42, 47, and 60%) comparable to classical chemotherapeutics for TNBC, but with lower side effects [114].

Imidazole-benzothiazole is a promising G4-binding motif. Taking this into consideration, IZTZ-1 (**45**—Figure 11) was developed and studied. It showed, in vitro, strong binding to c-MYC G4 (ΔTm = 15 °C) and inhibitory activity regarding melanoma cell growth (IC_50_ = 2.2 µM). Furthermore, a dual luciferase reporter assay and a flow cytometry assay showed that **45** could downregulate *c-MYC* transcription and expression and induce apoptosis. Moreover, it also inhibited tumor growth in a breast cancer xenograft mouse model [115].

The indolizinone **46** in Figure 11 was shown via fluorescence titration to selectively recognize MYC G4 (Ka = 9.9 × 10^5^). This compound can also downregulate the transcription of *c-MYC* in two different cancer cell lines with different translocation break points within the *c-MYC* (human non-small cell line A549 and human laryngeal epithelial cell line—Hep2) [116]. The 7-azaindole-1-carboxamide derivative **47** (Figure 11) was investigated as a dual G4 binder/PARP inhibitor. The study showed that the compound stacks on the G4-tetrad trough *π*-*π* interactions, forming a 2:1 ligand/G4 complex with Ka = 10^6.1^ M^−1^ [117]. Moreover, this compound also presents antiproliferative activity in HCC1937 cell lines, with an IC^50^ of 19.4 µM. In an in vivo assay, Compound **47** reduced the tumor volume by 35% in a MX1 cell line through the administration of 100 mg/kg every 2 days for 2 weeks [118].

**Figure 12:**
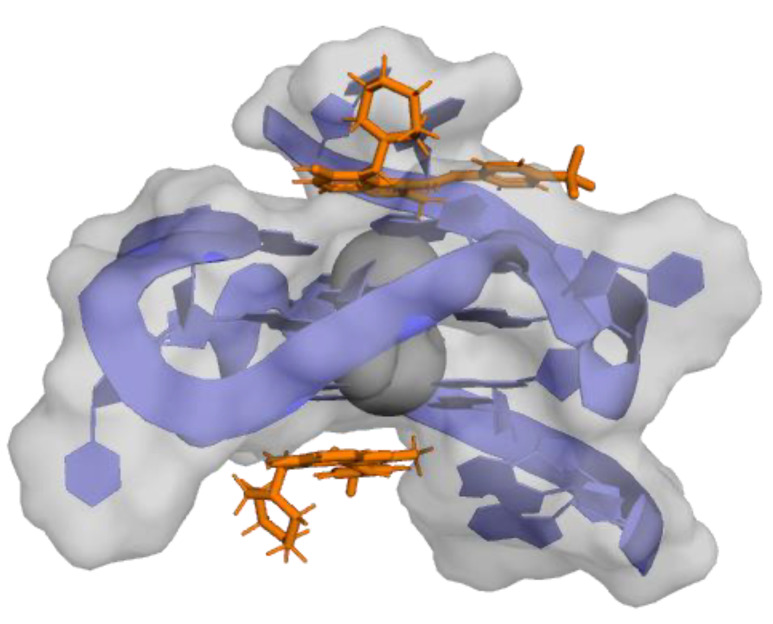
Schematic representation of Compound **49** (colored sticks) in a 2:1 complex with a *c-MYC* G4 structure (PDB ID: 5W77).

Compound D089 (**48**—Figure 11) is a benzofuran derivative discovered through an investigation using a focused library of “drug-like” small molecules binding c-MYC G4 sequences [119]. It has a preference to bind to c-MYC G4 over other G4s in oncogenes promoters and duplex DNA, and it binds through a reversible interaction, as observed using a sensorgram. Furthermore, this compound showed better antiproliferative activity against myeloma cells (IC_50_ = 5.8 µM) than the well-studied G4 binder BRACO-19 (IC_50_ = 15.3 µM) [120]. Another study discovered DC-34 (**9**—Figure 11), a benzofuran derivative very similar to **48** that can also selectively inhibit *c-MYC* transcription by binding to the G4 in its promoter. It exhibited that a ΔTm of 7.5 °C forms a 2:1 complex, stacking over two guanines at each terminal G-tetrad plane, with reconfigured segments capping the ligand, as shown in Figure 12. This compound presents better antiproliferative activity against myeloma cells (IC_50_ of 3.4 µM) than Analog **48 [121]**.

### 4.5. Flexible G4 Ligands

**Figure 13:**
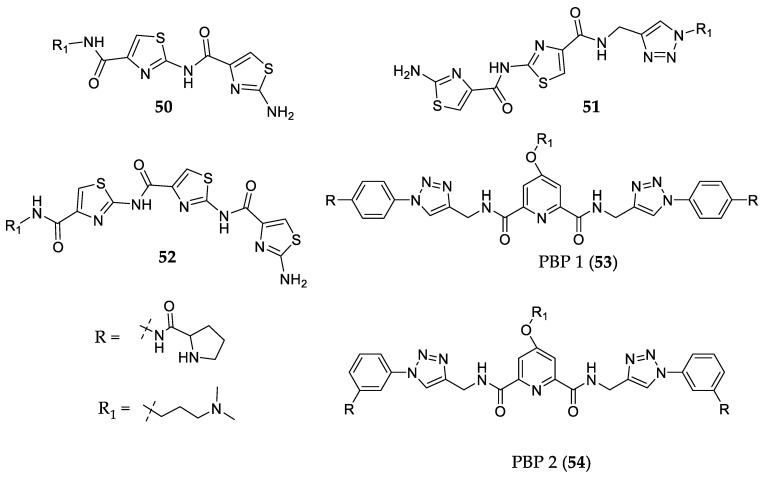
Structure of flexible c-MYC G4 ligands **50**–**54**.

Thiazoles are present in several anticancer compounds [122] such as bleomycin and tiazofurin [123]. The thiazole moiety can be used in polyamide compounds as shown by Dutta et al. [122]. These researchers synthesized three thiazole polyamides (**50**, **51,** and **52**—Figure 13), and the latter compound (**52**) was shown to be more selective to c-MYC G4 than to other gene promoter G4s and duplex DNA, binding strongly when compared to the other two polyamide derivatives of **50** and **51**. Compound 53 also showed the best antiproliferative activity against cancer cells compared to Compound **50** (IC_50_ values of 3.8 µM and 17.6 µM, respectively), but both showed insignificant cytotoxicity for non-malignant cells (IC_50_ *>* 50 µM).

Debnath et al. [124] developed two peptidomimetic congeners that were able to bind to and stabilize G4 structures. A FRET melting assay showed that PBP1 (**53**) and PBP2 (**54**) (Figure 13) have high selectivity for c-MYC G4 over duplex DNA and a thiazole orange displacement assay demonstrated that **53** and **54** have good affinities to different G4s, with EC_50_ values regarding c-MYC G4 of 8.5 µM and 1.3 µM, respectively. They also investigated the growth-inhibiting activities of compounds using an MTT assay. Compound **53** showed higher values of IC_50_ than **54** (17.9 and 3.3 µM, respectively). Afterwards, other authors also studied these compounds and noticed that **53** presented a higher dissociation constant (11.2 µM) than **54** (1.42 µM) via isothermal titration calorimetry studies, indicating therefore that **54** is a better binder of c-MYC G4. These authors also analyzed the preference of binding to c-MYC G4 and to BCL2 G4, concluding that **54** is selective toward c-MYC G4 whereas **53** does not show a significant preference. Both compounds presented potent cytotoxic activity in MCF-7 breast cancer cells (IC_50_ = 3.8 and 7.1 µM), moderate cytotoxicity for other cancer cell lines, and low cytotoxicity for normal cells [125].

### 4.6. Metal Complexes

**Figure 14:**
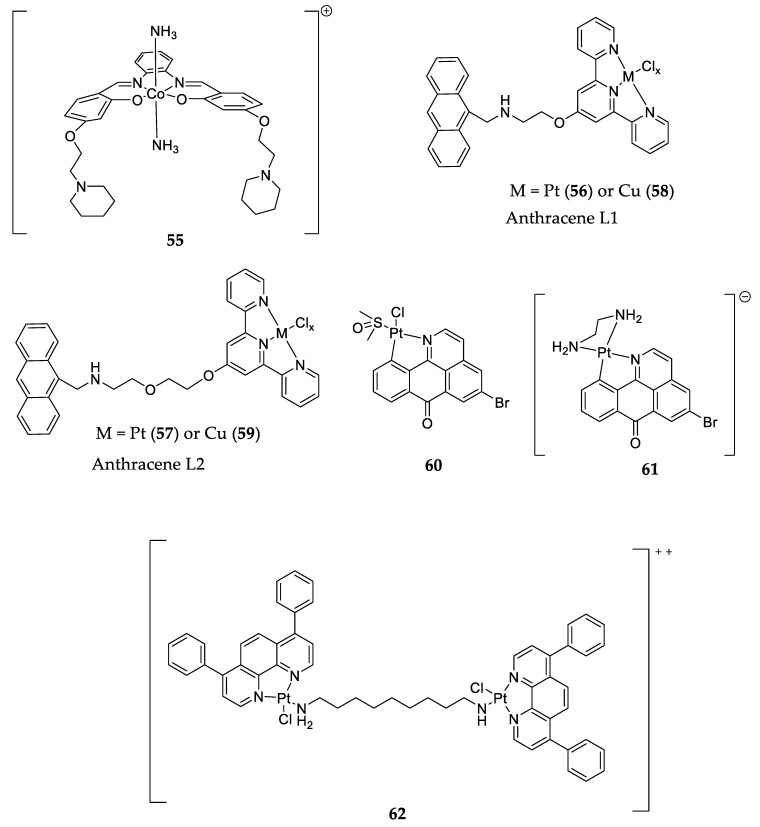
Structure of metal complexes able to bind to and stabilize c-MYC G4.

At the beginning of this century, the first G4 binders based on square planar metal complexes were reported. Since then, hundreds of new metal complex derivatives binding to G4 have been identified. Square-planar complexes with Ni II, Cu II, Co III, and Pt II are selective toward G4 over duplex DNA [24,126,127]. Co III compounds with NH_3_ as the coordinated ligand (**55**—Figure 14) have a higher affinity to c-MYC G4 than other complexes. The proposed binding mechanism considers that the NH_3_ groups establish hydrogen bond interactions with guanine’s oxygen atoms and that the salphen ligand displays *π*-*π* stacking interactions with the G-tetrad [126].

The complexes of Cu II and Pt II anthracene-containing terpyridine ligands (PtL^1,2^—**56** and **57**; and CuL^1,2^—**58** and **59**; Figure 14) are selective toward G4 over duplex DNA. In one study, the Pt complexes showed better G4 stabilization values in a FRET melting assay when compared to the other complexes or the ligands without complexation with metals. Furthermore, Pt complexes presented a higher affinity to c-MYC G4 than the Cu complexes. The authors also concluded that the compounds with bigger linkers (L^2^—**57** and **59**) had more affinity to bind to G4 than the smaller ones (**56** and **58**), as can be seen by the ΔTm values in Table 1 [128].

Other platinum complexes [Pt(L)(DMSO)Cl] (**60**) and [Pt(L)(pn)]Cl (**61**)) with 5-bromo-oxoisoaporphine as ligands (Figure 14) were studied for their ability to downregulate *c-MYC* expression through binding to its promoter G4. Complex **62** inhibited 99.9% of c-MYC protein levels and it also showed better selectivity to *c-MYC* in an FID assay than Complex **60**. Their cytotoxicity was evaluated via an MTT assay, and it was reported that the IC_50_ values varied between 5.1 and 31.1 µM for different cancer cell lines, but they showed very low cytotoxicity for non-malignant cells [129].

Dinuclear platinum complexes like **62** (Figure 14) were also shown to be able to stabilize G4 structures and bind more selectively to *c-MYC* G4 than to duplex DNA, with a ΔTm of 8.5 °C in a FRET melting assay. These complexes were able to interact with G-tetrads via *π* stacking and cross-linking with purines in G4 through their alkyl chains [130].

## 5. Conclusions

The *c-MYC* proto-oncogene encodes for a transcription factor whereby expression is deregulated in many cancer types. For this reason, several strategies have been used in the past years to, directly or indirectly, target c-MYC but, so far, these attempts have been unsuccessful. Another approach being studied aims to target the expression of *c-MYC* in cancer cells via the stabilization of the G4 structures present in its promoter region.

Table 1 summarizes the major achievements made so far. It can be concluded that several small molecules with different chemotypes and shapes have been investigated, and these compounds have a core with differently organized aromatic rings to better interact with G-quartets and stabilize the G4. Interestingly, all of the molecules reaching clinical trials (**13, 14,** and **16**) have four fused aromatic rings and are potent G4 stabilizers, but they are not selective toward c-MYC G4. On the other hand, some c-MYC G4 ligands with two fused aromatic rings and consequently being more flexible were shown to have a preference of binding to this G4, and also showed good anticancer activity in vitro and in vivo (**44** and **45**). Moreover, it is among this class of compounds that we can find G4 ligands that are believed to act in cancer cells by targeting the G4-binding proteins or the interaction between the c-MYC G4 and the protein, such as the quinazolines **42** and **43**. In fact, research into the G4-protein interactome is emerging and it is believed that targeting these interactions with small molecules may be the solution to achieve the desired selectivity toward a certain G4 structure.

Overall, this review has put into evidence all of the efforts that have been made to target c-MYC G4 and the new approaches being explored, and has highlighted several lead compounds that can be used in further investigations.

## Figures and Tables

**Table 1:** Summary of best c-MYC G4 ligands’ characteristics.

		Binding to c-MYC G4			Anticancer Activity	
Group	N°	Strength (Kd, Ka, ΔTm)	Preference for c-MYC G4 (Yes/No)	In Vitro	In Vivo	Ref.
Macrocycles	1		No	Reduces telomerase activity. Decreases *c-MYC* and *hTERT* expression. Inhibits cancer cell growth.	Inhibits tumor growth in two xenograft tumor models.	[55,56]
	3	ΔTm = 17.2 °C	No	Decreases *c-MYC* expression. Decreases Pif1 helicase activity. Cytotoxicity activity (IC_50_ < 0.01 µM) in MCF-7 cells.		[58,59]
Ligands with four or more fused aromatic rings	4		Yes	Decreases *c-MYC* expression. Antiproliferative activity in cancer cells (IC_50_ = 0.24–4.8 µM).		[63]
	5	ΔTm = 26.6 °C	__	Decreases *c-MYC* expression. Cytotoxicity activity (IC_50_ of 4.7 µM) in Burkitt’s lymphoma (RAJI) cell line.	Inhibits tumor growth in a Burkitt’s lymphoma xenograft model.	[61]
	6 7	ΔTm = 7 °C ΔTm = 17 °C	No	Cytotoxicity activity (IC_50_ 2.3–3.1 µM) in RAJI cell lines.		[64]
	8 9 10	ΔTm = 22.0 °C ΔTm = 16.6 °C ΔTm = 13.7 °C	Yes	Decrease *c-MYC* expression. Cytotoxicity activity (IC_50_ = 0.02–5.53 µM) for different cancer cell lines.	Compound 10 significantly decreases tumor growth in a lung cancer xenograft model.	[65]
	12	ΔTm = 21 °C	__	Decreases *c-MYC* expression.		[69]
	13	ΔTm > 15 °C	__	Specific toxicity against BRCA1/2-deficient cells. Inhibits RNA-polymerase I activity and the formation of nucleolin/G4 complexes. Decreases *c-MYC* expression and induces apoptosis.	Reached Phase II clinical trial.	[73,76]
	14	ΔTm > 15 °C	No	Specific toxicity against BRCA1/2-deficient cells. Inhibits RNA polymerase I and Topoisomerase II. Induces G4-mediated DNA damage.	Phase I clinical trial.	[81,131]
	15	ΔTm = 4.4 °C	__	Decreases *c-MYC* expression. Cytotoxicity activity (IC_50_ = 1.1 μM) in CNE-1 cells.	Inhibits tumor growth of CNE-1 cells in zebrafish xenograft model.	[82]
	16	-	No	Decreases *c-MYC* expression in mRNA. Cytotoxicity activity in different cell lines. ∙	Phase I clinical trial for acute myelogenous leukemia.	[83,85,86]
	17 18	ΔTm ~ 10 °C	Yes	Inhibits lymphoma cell growth.		[87]
	19	ΔTm = 16.4 °C	No	Decreases the expression of *c-MYC* and *BCL-2*. Causes telomere uncapping. Cytotoxicity activity in different cell lines.	Inhibits tumor growth in a metastatic lymph node cell in mice xenograft model.	[88,89]
	20	ΔTm > 6 °C	No	Decreases the expression of c*-MYC* and *HIF1α*.	Inhibits tumor growth in a colon cancer mouse model.	[91]
	21	ΔTm = 29 °C	Yes	Decreases *c-MYC* expression. Cytotoxicity activity in NCI cells (IC_50_ = 4 µM).		[92]
Ligands with three fused aromatic rings	22		Yes	Decreases *c-MYC* level by 25–40% in Ramos cells.	-	[95]
	24	ΔTm = 12.8 °C	No	Decreases *c-MYC* and *BCL2* expression. Cytotoxicity activity in Jurkat human T lymphoblastoid cells (IC_50_ = 17.0 µM).		[96]
	26	ΔTm = 20 °C	Yes	Decreases *c-MYC* expression. Cytotoxicity activity in different cancer cell lines (IC_50_ = 2.1–4.2 µM).	Inhibits tumor growth in cervical squamous cancer in nude mice xenograft model.	[97]
	27 28	ΔTm = 4 °C ΔTm = 1.9 °C	Yes	Decreases *c-MYC* expression. Cytotoxicity activity in different cancer cell lines.		[98,99]
	29	ΔTm= 15.8 °C	Yes	Cytotoxicity activity in HeLa (IC_50_ = 3.4 µM) and in HCT116 (IC_50_ = 3.2µM) cells.		[100]
	31	Kd = 0.17 µM	__	Decreases *c-MYC* expression. Cytotoxicity activity in HCT116 cells (IC_50_ = 2.1 µM).		[102]
	34	ΔTm = 23.4 °C	__	Decreases *c-MYC* expression. Cytotoxicity activity in HeLa (IC_50_ = 2.5 µM) and in A549 (IC_50_ = 6.4 µM) cells.		[103]
	37	Kd = 36 nM	Yes	Decreases *c-MYC* expression.		[104]
	38	ΔTm = 15.0 °C	__	Decreases *c-MYC* expression. Downregulates RNA polymerase I transcription. Antiproliferative activity in different cancer cell lines.		[107]
Ligands with two fused aromatic rings	39	ΔTm = 23.7 °C	__	Decreases *c-MYC* expression.	Inhibits tumor growth in liver cancer in nude mouse xenograft model.	[108]
	40	ΔTm = 9 °C	__	Decreases *c-MYC* expression. Showed good binding affinity to NM23-H2 protein (K_D_ = 5.29 µM).		[109]
	41 42	ΔTm = 12.1 °C ΔTm = 12.9 °C	__	Decrease *c-MYC* expression. Interfere with the binding of c-MYC G4 with NM23-H2.	Inhibit proliferation of SiHa cells in a dose-dependent manner.	[110]
	43	Binding affinity to NM23-H2 protein (K_D_ = 3.1 μM)	No	Decreases *c-MYC* expression by disrupting the interaction between NM23-H2 and c-MYC G4. Induces cell cycle arrest and apoptosis. Cytotoxicity activity in different cancer cells.	Inhibits tumor growth in a cervical squamous cancer xenograft mouse model.	[111]
	44	*K*_D_ = 1.3 μM	Yes	Decreases *c-MYC* expression. Cytotoxicity activity in different cancer cells (IC_50_ = 0.7–0.9 µM).	Inhibits tumor growth in a breast cancer xenograft mouse model.	[114]
	45	ΔTm = 15 °C	Yes	Decreases *c-MYC* expression. Cytotoxicity activity in melanoma cells (IC_50_ = 2.2 µM). Induces apoptosis.	Inhibits tumor growth in a melanoma mouse model.	[115]
	46	Ka = 9.9 × 10^5^ M^−1^	Yes	Decreases *c-MYC* expression.		[116]
	47	Ka = 10^6.1^ M^−1^	No	Acts as dual G4 binder/PARP inhibitor. Cytotoxicity activity in HCC1937 cells (IC_50_ = 19.4 µM).	Inhibits tumor growth in a breast cancer xenograft mouse model.	[117,118]
	48		Yes	Decreases c-*MYC* expression. Antiproliferative activity in different myeloma cell lines (IC_50_ = 5.8 µM).		[119,120]
	49	ΔTm = 7.5 °C	Yes	Decreases *c-MYC* expression. Cytotoxicity activity in myeloma cells (IC_50_ =3.4 µM).		[121]
Flexible ligands	52	ΔTm = 22 °C	Yes	Decreases *c-MYC* expression. Cytotoxicity activity in HeLa cells (IC_50_ = 3.8 µM) and in A549 cells (IC_50_ =3.2 µM).		[122]
	53 54	ΔTm = 15.0 °C ΔTm = 6.0 °C	No	Decrease *c-MYC* expression. Cytotoxic activity in MCF-7 cells (IC_50_ = 3.80 and 7.1 µM, respectively).	-	[124,125]
Metal complexes	56 57 58 59	ΔTm = 10.2 °C ΔTm = 15.6 °C ΔTm = 2.0 °C ΔTm = 7.3 °C	No	Decrease *c-MYC* expression.		[128]
	60 61		__	Decrease *c-MYC* expression. Cytotoxic activity in Hep-G2 cells (IC_50_ = 10.1 and 5.1 µM, respectively).		[129]

## Data Availability

Not applicable.

## Abbreviations

AURKA: Aurora Kinase A; BRD4: Bromodomain-containing protein 4; CDK: Cyclin-dependent kinase 7; CPEB: cytoplasmic polyadenylation element binding; GLS: glutaminase; mTOR: mammalian target of rapamycin; PLK1: Serine/threonine protein kinase; USP: ubiquitin-specific peptidase.
